# The three-dimensional influence and clinical significance of anterior traction with rapid expansion during dental replacement

**DOI:** 10.2340/aos.v84.44580

**Published:** 2025-08-27

**Authors:** Naizheng Gou, Xiaoqin Wang, Haiyan Li, Zuoying Dong

**Affiliations:** a Department of Stomatology, Qingdao Jiaozhou Central Hospital, Jiaozhou, China; b Department of Medical Insurance, Qingdao Jiaozhou Central Hospital, Jiaozhou, China

**Keywords:** Tooth malformation, orthodontics, rapid maxillary expansion, Hyrax-type

## Abstract

**Objectives:**

This study aimed to investigate the effects of rapid maxillary expansion using a Hyrax-type appliance (RME-Hyrax) with anterior traction on the maxillary arch during mixed dentition and its clinical significance, focusing on changes in arch dimensions, occlusal stability, and masticatory function.

**Materials and methods:**

Forty-two patients with transverse maxillary deficiency were treated with RME-Hyrax, while an untreated control group of 40 received no treatment. Pre- and post-treatment dental casts were analyzed using a 3D scanner. Arch length, width, and occlusal stability were measured. Statistical analysis was performed using paired t-tests and linear regression.

**Results:**

RME significantly increased maxillary arch widths at all measured points: 3CW: +4.06 mm, 4CW: +4.88 mm, 5CW: +4.09 mm, 6MCW: +3.00 mm (all *p* < 0.001). Mandibular arch widths similarly increased (3CW: +3.18 mm, 4CW: +4.00 mm, 5CW: +4.61 mm, 6MCW: +3.07 mm; all *p* < 0.001). Buccal movement of first permanent molars was significant in both arches (maxillary: 1.61–1.78 mm, mandibular: 1.52–1.68 mm; all *p* < 0.001). Occlusal stability improved clinically, evidenced by increased maximum bite area (+19.66 mm², exceeding the 15% threshold for functional gain; *p* < 0.001) and force (+1.06 kg, surpassing the 0.5 kg minimum meaningful change; *p* < 0.001), with reduced asymmetry index (-22.03%, *p* < 0.001). Masticatory efficiency improved from 54.22 to 84.61% (Δ30.39%, < 25% threshold; *p* < 0.001).

**Conclusions:**

Hyrax-type RME is effective for correcting transverse maxillary deficiencies, expanding both the maxillary and mandibular arches, and improving occlusal and masticatory function. Early intervention can significantly enhance dental and functional health.

## Introduction

Children typically begin to lose their primary teeth around the age of six, with permanent teeth gradually replacing these baby teeth. This process represents a significant and intricate phase of oral development, known as the dental replacement stage or mixed dentition stage [1, 2]. During the mixed dentition period, the jaw undergoes growth, the roots of the deciduous teeth are progressively absorbed, leading to their eventual loss, while the permanent teeth erupt from beneath the primary teeth to take their place [3]. Mixed dentition is a crucial time for both tooth and jaw development and presents one of the prime opportunities for orthodontic intervention [4, 5]. Throughout the tooth replacement process, alterations in jaw volume, shape, and spatial positioning may arise due to abnormal growth and development, whether congenital or acquired. These changes can result in an improper denti- maxillary relationship, dysfunction within the oral system, and atypical facial morphology, collectively referred to as denti- maxillofacial deformities [6, 7]. The prevalence of odontomaxillofacial deformities is relatively high in China [8]. Such deformities not only affect facial esthetics but also negatively influence facial development, as well as chewing, swallowing, and sleep patterns [9–12]. When the relationship between the dental arch and the jaw is misaligned, it can disrupt the normal development of facial bones and soft tissues, hinder the dental arch’s ability to occlude properly, and impair chewing and swallowing functions, ultimately affecting overall digestion and nutrient absorption [13]. Furthermore, odonto-maxillofacial deformities can impose significant psychological burdens on children and adolescents, adversely impacting their physical and mental health development [14].

Malocclusion is a common type of odontomaxillofacial malformation that occurs in children and adolescents, primarily where anterior occlusion during growth can lead to abnormal jaw growth. The World Health Organization recognizes malocclusion as one of the three major oral diseases, affecting approximately 260 million children in China [8]. This condition can significantly impact children’s oral function, oral health, and the growth of teeth and the maxillofacial structure, necessitating timely correction upon diagnosis. Maxillary transverse deficiency (MTD) is a common clinical manifestation of malocclusion, associated with both genetic and environmental factors. It is primarily characterized by maxillary arch stenosis, dental crowding, a high palatal arch, and posterior crossbite [15]. MTD can result in respiratory and swallowing dysfunctions, including increased resistance in the nasal passages, narrowing of the pharyngeal airway, and abnormal swallowing patterns. Maxillary expansion is a standard treatment for patients with MTD, which can be categorized into fast expansion and slow expansion based on the frequency of applied force [16–19].

Rapid maxillary expansion using a Hyrax-type appliance (RME-Hyrax) is a conventional treatment method for maxillofacial dental disorders, effectively addressing the respiratory and swallowing dysfunction associated with these conditions [20, 21]. Commonly employed expansion devices for RME include dental support and hybrid support types [22]. The Hyrax-type appliance exemplifies the dental support type used in RME-Hyrax; unlike the mixed support type diffuser, it lacks an acrylic acid base, allowing it to exert force directly on the anchorage teeth without causing compression damage to the mucosa. Conversely, the Haas type deamer represents the hybrid support type, utilizing teeth, periodontal tissue, and the palate for anchorage, and delivering corrective force through an acrylic base, thereby facilitating the expansion of the palatal midsuture and improving MTD outcomes [23–25]. Patients with MTD frequently exhibit a disharmony between the mandibular arch and basal bone width [26–28]. While the therapeutic efficacy of the Hyrax-type diffuser in treating MTD patients has been established, its potential impact on the mandibular dental arch during treatment remains uncertain. To address this problem, the present study measured the maxillary and mandibular arches of adolescents before and after treatment with the RME-Hyrax appliance to explore its possible therapeutic effect on the mandibular dental arch and maxillary traction.

## Materials and methods

### Research objects and groups

This study was approved by the Medical Ethics Committee of Qingdao Jiaozhou Center Hospital (approval number: 20240925-049). Participants were selected from patients treated for maxillary lateral defects in the Oral Orthodontics Department of our hospital between August 2022 and August 2024. The experimental group comprised 42 patients with mixed dentition who were treated using a Hyrax-type appliance for RME-Hyrax, including 21 males and 21 females. Their ages ranged from 6.00 to 9.00 years, with a mean age of 7.46 years and a mean treatment duration of 11 months. The untreated control group consisted of 40 patients who did not receive any orthodontic treatment, matched to the experimental group by chronological age (± 6 months), sex distribution (*p* = 0.650), and dental development stage (assessed via panoramic radiographs using the Demirjian index). Baseline maxillary and mandibular arch measurements showed no significant intergroup differences (all *p* > 0.05, see Tables 2–3 T0 data). This study followed a nonrandomized parallel cohort design, where the experimental group received active intervention while the untreated control group received no orthodontic treatment. The sample size was determined based on clinical feasibility, ensuring sufficient power to detect clinically significant changes (≥ 2 mm arch width difference with α = 0.05 and β = 0.20). All patients and their families provided informed consent prior to participation in the study.

**Table 1:** Arch length of mandible (before treatment: T0, after treatment: T1, X ± SD, the same below).

Measurement Index		T0 (mm)	T1 (mm)	*p*
Untreated control group	Anterior arch length	3.35 ± 0.64	3.37 ± 0.42	0.803
	Middle arch length	7.31 ± 0.95	7.32 ± 0.69	0.870
	Posterior arch length	18.23 ± 0.31	18.23 ± 0.42	0.453
Treatment group	Anterior arch length	3.36 ± 0.62	4.67 ± 1.08	< 0.001
	Middle arch length	7.12 ± 0.18	7.33 ± 0.34	0.648
	Posterior arch length	18.20 ± 0.31	18.22 ± 0.53	0.803

**Table 2:** Maxillary central incisor eruption status.

Parameter	Untreated control group		Treatment group	
	T0	T1	T0	T1
Crown height (mm)	5.30 ± 0.39	5.51 ± 0.42	5.22 ± 0.41	7.04 ± 0.38*
Eruption stage (%)				
Nolla 7 (1/3 crown erupted)	100	40	100	0
Nolla 8 (2/3 crown erupted)	0	60	0	83.30
Nolla 9 (full eruption)	0	0	0	16.70

* *p* < 0.001 vs T0; Data presented as X± SD

Note: Nolla stages classification: Stage 7 = 1/3 crown erupted, Stage 8 = 2/3 crown erupted, Stage 9 = full eruption.

**Table 3:** The width of upper and lower dental arches in the untreated control group.

Measurement index		T0 (mm)	T1 (mm)	*p*
Maxillary	3CW	31.72 ± 0.87	31.78 ± 0.79	0.470
	4CW	38.93 ± 0.88	38.95 ± 0.87	0.554
	5CW	44.50 ± 1.43	44.50 ± 1.35	0.952
	6MCW	52.60 ± 1.58	52.59 ± 1.62	0.947
Mandible	3CW	24.72 ± 0.87	24.76 ± 0.85	0.095
	4CW	31.53 ± 0.88	31.53 ± 0.84	0.947
	5CW	36.89 ± 1.04	36.93 ± 1.04	0.066
	6MCW	45.52 ± 1.15	45.70 ± 1.50	0.325

### Inclusion criteria

1. This study focuses on mixed dentition patients, aged 6 to 12 years, who present with lateral maxillary defects.
2. The general health of these patients is satisfactory, with the first permanent molar having erupted prior to treatment, and no signs of loosening or severe caries.
3. The experimental group received treatment using the RME-Hyrax appliance, without the use of any additional devices during the treatment process.
4. The RME-Hyrax appliance is key when treating skeletal Class III malocclusion, especially when used during the mixed dentition phase (i.e. when deciduous teeth and permanent teeth co-exist), approximately between the ages of 8–12 years. At this stage, the maxillary growth potential is greater, and the expansion effect is better.

### Exclusion criteria

1. Poor general health, a family history of immune system disorders, or mental illness;
2. Patients with congenital lip and palate clefts;
3. A history of orthodontic treatment, maxillofacial trauma, or surgery;
4. Congenital or acquired loss of deciduous teeth or permanent teeth.

### Treatment plan

All subjects in the experimental group received treatment with a fixed RME-Hyrax appliance attached to the first maxillary permanent molar. The Hyrax expander was activated twice daily (0.25 mm per turn) for 2–3 weeks until achieving target expansion (5–10 mm). After active expansion, the appliance was retained for 3–6 months to consolidate new bone formation.

#### Anterior traction protocol

Following the completion of RME and a 3-month retention period, a facemask (Delaire-type) was attached to the RME-Hyrax appliance via elastic chains. Traction force (300–500 g per side) was applied bilaterally in a downward and forward direction (~30° to the occlusal plane). Patients were instructed to wear the facemask for 12–14 h per day throughout the active traction phase. The specific force magnitude was adjusted based on the patient’s age, skeletal maturity and tolerance and monitored at 4-week intervals.

Fixed appliance therapy (preadjusted edgewise) was subsequently initiated and maintained for 6–9 months to achieve final occlusion. The reported mean total treatment duration of 11 months (range: 9–14 months) comprised three phases: active expansion (2–3 weeks), retention (3–6 months), and fixed appliance therapy (6–9 months). Dental casts were obtained pre-treatment and post-treatment (after completion of all orthodontic phases) to measure arch dimensions and molar movements. The untreated control group received no intervention.

### Test items

The dental molds obtained prior to treatment and following the removal of the pedicle diffuser were processed using a 3D scanner (3Shape D250 laser, DK). Digital landmarks were tracked with VAM software (Canfield Scientific Inc., Fairfield-NJ, USA), establishing a coordinate system based on the origin of the former nasal spine point. The X-axis extended through the anterior nasal spine point and was parallel to the left and right infraorbital points; the Y-axis passed through both the root nasal spine point and the anterior nasal spine point; and the Z-axis extended through the anterior nasal spine point, remaining parallel to the orbital ear plane. Measurements were taken for the length of the dental arch, the width between teeth, and the changes in the buccal and lingual directions of specific teeth before and after the removal of the pedicle diffuser. Measurements were categorized as follows: before treatment (Stage T0) and after treatment (Stage T1).

(1) Measurement of arch length (AL): Anterior arch length refers to the vertical distance between the contact point of the central incisor and the junction of the tips of both deciduous teeth, denoted as ALa. The middle arch length is defined as the vertical distance between the junction of the cusps of both deciduous teeth and the connection between the fovea of the first deciduous molars, represented as ALm. Posterior arch length is the vertical distance between the fovea line of the bilateral first deciduous molars and the fovea line of the bilateral first permanent molars, indicated as ALp.

(2) Measurement of the width of the horizontal arch formed by the tips of the maxillary and mandibular teeth. The English abbreviations for the measurement markers of the maxillary and mandibular teeth are as follows:

**Table:** 

Measuring marker	Implication
U3	Maxillary deciduous fangs
U4	Maxillary first deciduous molar
U5	Maxillary second deciduous molar
U6	Maxillary first permanent molar
L3	Mandibular deciduous fangs
L4	Mandibular first deciduous molar
L5	Mandibular second deciduous molar
L6	Mandibular second deciduous molar

The horizontal arch width of the cusp is expressed by CW, and the measurement indicators are as follows:

**Table:** 

Measurement index	Implication
3CW	The distance between the cusps of both deciduous teeth
4CW	The distance between the buccal tips of the first molar teeth
5CW	The distance between the mesiobuccal tips of the second mastomolar teeth on both sides
6MCW	The distance between the mesiobuccal tips of both first permanent molars

(3) Measurement of changes in the buccal and lingual direction of the upper and lower mandible, the English abbreviations of each measurement marker are as follows:

**Table:** 

Measuring marker	Implication
U3c	Central point of maxillary deciduous tooth crown
U3r	Maxillary cuspidate root center
U6c	Central point of maxillary first permanent molar crown
U6r	Central root point of maxillary first permanent molar
L3c	Central point of crown of maxillary deciduous cusp
L3r	Central root point of maxillary deciduous cusp
L6c	Central point of the mandibular crown of the first permanent molar
L6r	Central root point of mandibular first permanent molar

The difference between T0 and T1 is characterized by buccolingual variation. In the right quadrant, a negative value of T0–T1 signifies buccal movement, while a positive value indicates lingual movement. Conversely, in the left quadrant, a positive T0–T1 value denotes buccal movement, whereas a negative value indicates lingual movement.

(4) Evaluation of occlusal stability and masticatory function. The occlusal function was assessed before and after treatment using the T-scan III occlusal analysis system (Tekscan Inc., Boston, MA, USA), which measured the maximum occlusal area, maximum bite force, and bite force asymmetry index. The asymmetry index was calculated as follows:


$$\text{Asymmetry index }(\text{\%})=\frac{\sum \left|{\text{F}}_{\text{left}}-{\text{F}}_{\text{right}}\right|}{{\text{F}}_{\text{total}}}\times 50$$

where F_left_ and F_right_ represent the total occlusal force (kg) on left/right quadrants, and F_total_ is the sum of bilateral forces. A value < 25% indicates balanced occlusion. Additionally, masticatory efficiency was evaluated pre- and post-treatment to determine any improvements in the patients’ masticatory function. Masticatory efficiency was calculated using the formula: Chewing efficiency = (total weight of chewed food – total weight of remaining food scraps)/total weight of chewed food * 100%.

(5) Assessment of incisor eruption status: Crown height (CH): Vertical distance from cementoenamel junction to the incisal edge of maxillary central incisors, measured on digital models. Eruption stage: Classified using Nolla’s stages (Stage 7: 1/3 crown erupted; Stage 8: 2/3 crown erupted; Stage 9: full eruption). Measurements performed at T0 and T1 by two calibrated examiners (ICC > 0.85).

### Statistical methods

All model data were measured three times and analyzed using GraphPad Prism 8 (v8.4.3). Continuous variables are reported as mean ± standard deviation (X ± S). To control for confounding factors, baseline matching was performed for age (± 6 months), sex (chi-square test), and dental development stage (Demirjian index). Paired t-tests were used to assess within-group changes (T0 vs. T1), while independent t-tests compared between-group differences in Δ values (T1–T0). Multivariable linear regression, adjusted for age and sex, was applied to all primary outcomes – including arch widths, molar movements, and bite parameters – with treatment group as the main predictor. Effect sizes were calculated using Cohen’s *d*, with *d* > 0.8 indicating a large effect. The correlation between maxillary and mandibular molar movements was assessed using Pearson’s linear regression, with statistical significance set at *p* < 0.05.

## Result

### RME-Hyrax treatment can significantly increase the length of the mandibular anterior dental arch and the width of each dental arch

**Figure 1:**
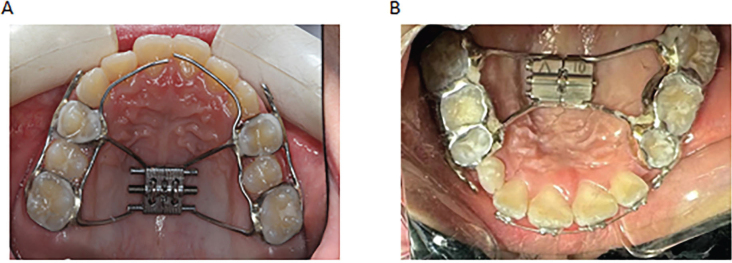
RME-Hyrax maxillary and mandibular extension method. (A) Hyrax RME maxillary expansion. (B) Hyrax RME mandibular expansion.

By measuring the anterior, middle, and posterior mandibular arch lengths, we found that the anterior mandibular arch length increased significantly following the intervention of RME-Hyrax (Figure 1) (*p* < 0.001), with an average increase of 1.31 mm. In contrast, no significant differences were observed in the middle and posterior arch lengths before and after treatment (Table 1).

The significant anterior arch length increase (ΔALa = 1.31 mm) coincided with active eruption of maxillary central incisors, evidenced by substantial crown height gain (T0: 5.22 ± 0.41 mm; T1: 7.04 ± 0.38 mm; Δ1.82 mm, *p* < 0.001) and clear eruption stage progression: while all subjects began at Nolla stage 7 (crown 1/3 erupted), post-treatment assessment revealed 83.3% advancing to stage 8 (2/3 erupted) and 16.7% reaching stage 9 (full eruption), whereas untreated control subjects showed minimal crown height change (Δ0.21 mm, *p* = 0.307) with only 60% progressing to stage 8 and 40% remaining at stage 7 (Table 2).

**Figure 2:**
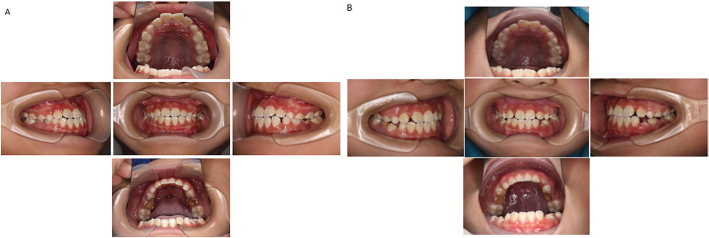
Length and width of maxillary and mandibular anterior arches before and after Hyrax RME treatment. (A) Length and width of maxillary and mandibular anterior arches before Hyrax RME treatment. (B) Length and width of maxillary and mandibular anterior arches after Hyrax RME treatment.

The maxillary arch widths of both the control and experimental groups were measured before and after treatment, specifically at 3CW, 4CW, 5CW, and 6MCW. Our data indicated that there was no significant difference in the maxillary arch width of the control group before and after treatment (*p* > 0.05). In contrast, the experimental group exhibited a significant increase in maxillary arch width following treatment (Figure 2), with average increases of 4.06 mm (Cohen’s *d* = 4.23) at 3CW, 4.88 mm (*d* = 3.52) at 4CW, 4.09 mm (*d* = 2.88) at 5CW, and 3.00 mm (*d* = 2.55) at 6MCW (all *p* < 0.001). Similarly, mandibular arch widths increased by 3.18 mm (*d* = 4.12) at 3CW, 3.18 mm (*d* = 4.12) at 3CW, 4.00 mm (*d* = 3.96) at 4CW, 4.61 mm (*d* = 3.80) at 5CW, and 3.07 mm (*d* = 3.09) at 6MCW (all *p* < 0.001) (Table 3). Conversely, the untreated control group demonstrated no significant changes (all *p* > 0.05). The > 3 mm arch expansion in the treatment group represents a 9–15% increase in arch width, exceeding the 2 mm minimum clinically important difference (MCID) for functional improvement in transverse deficiencies (Tables 2 and 3).

These results indicated that RME-Hyrax could not only widen the maxillary arch but also significantly increase mandibular arch width.

### RME-Hyrax treatment can facilitate the buccal movement of the first permanent molars in both the upper and lower jaws

By establishing a three-dimensional coordinate system, we identified the coordinates of the measurement markers corresponding to the first permanent molars in the upper and lower jaws, specifically U6c, U6r, L6c, and L6r.

**Figure 3:**
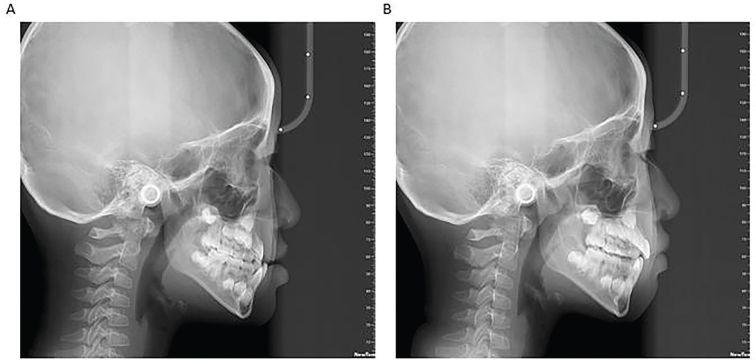
Changes of maxillary and mandibular first permanent molar before and after Hyrax RME treatment. (A) Hyrax RME before treatment of the maxillary first permanent molar. (B) RME-Hyrax after treatment of the maxillary first permanent molar.

Our results indicated that there was no significant change in the position of the maxillary and mandibular first permanent molars before and after treatment in the control group. In contrast, following treatment, the position of the first permanent molars in both the upper and lower jaws of the experimental group exhibited buccal movement (*p* < 0.001), (Figure 3). The specific movement values were as follows: Buccal movement of first permanent molars was significant in both jaws: left maxillary crown (1.78 mm, *d* = 1.28) and root (1.63 mm, *d* = 1.13); right maxillary crown (1.61 mm, *d* = 1.20) and root (1.61 mm, *d* = 1.18); left mandibular crown (1.68 mm, *d* = 1.10) and root (1.56 mm, *d* = 1.02); right mandibular crown (1.61 mm, *d* = 1.23) and root (1.52 mm, *d* = 1.13) (all *p* = 0.001). Similarly, the crown and root centers of the first permanent molar on the right side of the mandible moved 1.61 mm and 1.52 mm buccally, respectively. These findings suggest that Hyrax RME treatment can induce buccal movement of both maxillary and mandibular first permanent molars (Tables 4 and 5).

**Table 4:** The width of upper and lower dental arches in the treatment group.

Measurement index		T0 (mm)	T1 (mm)	*p*	Cohen’s d
Maxillary	3CW	31.8 ± 0.94	35.86 ± 0.96	< 0.001	4.23
	4CW	38.91 ± 1.31	43.79 ± 1.38	< 0.001	3.52
	5CW	44.61 ± 1.39	48.70 ± 1.42	< 0.001	2.88
	6MCW	52.53 ± 1.15	55.53 ± 1.18	< 0.001	2.55
Mandible	3CW	24.74 ± 0.59	27.91 ± 0.61	< 0.001	4.12
	4CW	31.45 ± 0.51	35.45 ± 0.50	< 0.001	3.96
	5CW	36.91 ± 0.83	41.52 ± 0.90	< 0.001	3.80
	6MCW	45.52 ± 0.97	48.59 ± 0.99	< 0.001	3.09

**Table 5:** Coordinates of the landmarks of the upper and lower first permanent molars in the untreated control group.

Measuring Marker	Measurement index	T0 (mm)	T1 (mm)	*p*
Left	U6c	23.20 ± 1.17	23.20 ± 1.19	0.999
	U6r	22.40 ± 1.41	22.40 ± 1.30	0.813
	L6c	23.10 ± 1.28	23.02 ± 1.26	0.431
	L6r	22.32 ± 1.36	22.40 ± 1.35	0.381
Right	U6c	-21.30 ± 1.30	-21.30 ± 1.28	0.999
	U6r	-21.23 ± 1.31	-21.25 ± 1.50	0.933
	L6c	-21.16 ± 1.40	-21.16 ± 1.26	0.999
	L6r	-21.09 ± 1.53	-21.10 ± 1.37	0.885

### Following RME-Hyrax treatment, a linear correlation was observed between the root centers of the first permanent molars in the maxillary and mandibular arches

**Figure 4:**
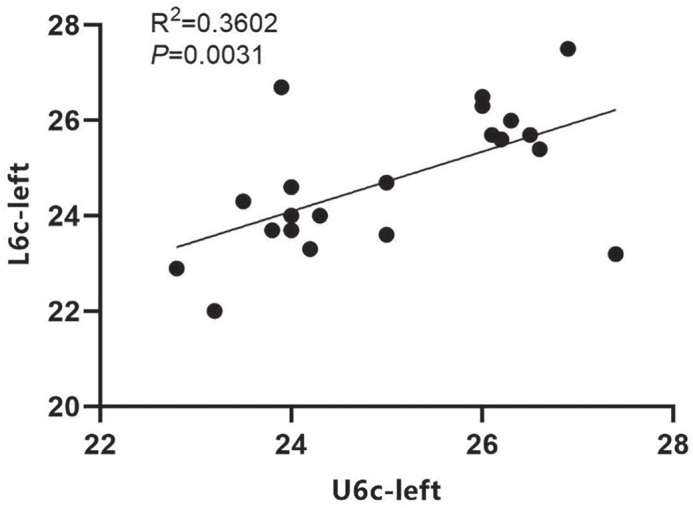
Correlation analysis of left U6c and L6c coordinates after treatment in the treatment group.

**Figure 5:**
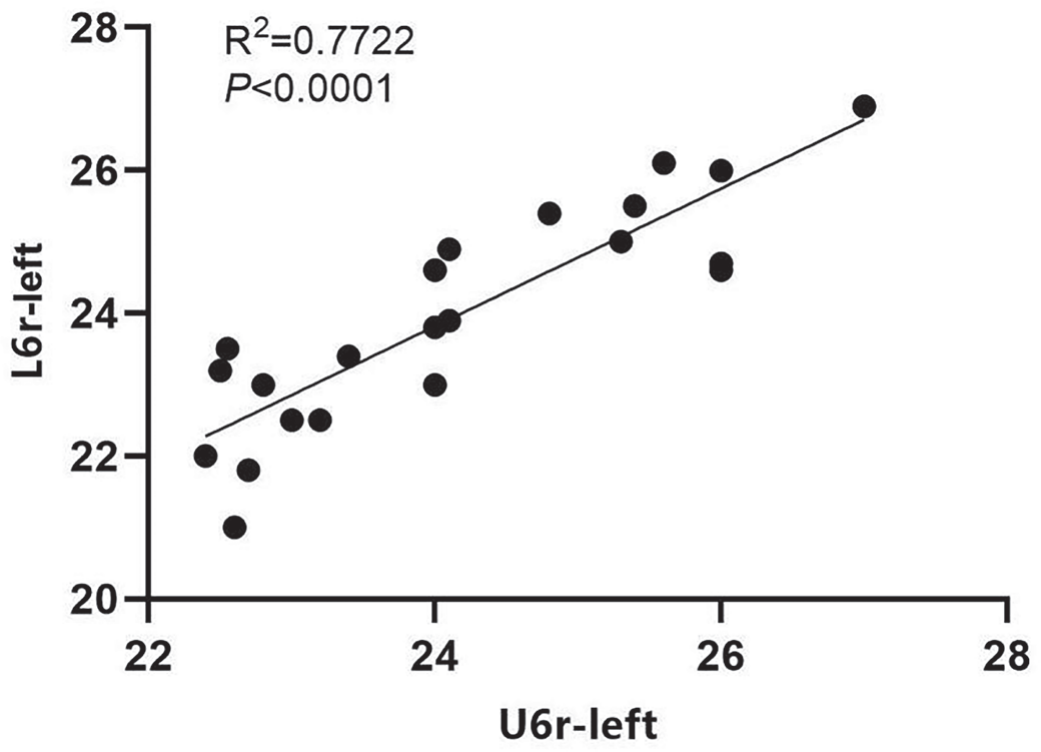
Correlation analysis of left U6r and L6r coordinates after treatment in the treatment group.

**Figure 6:**
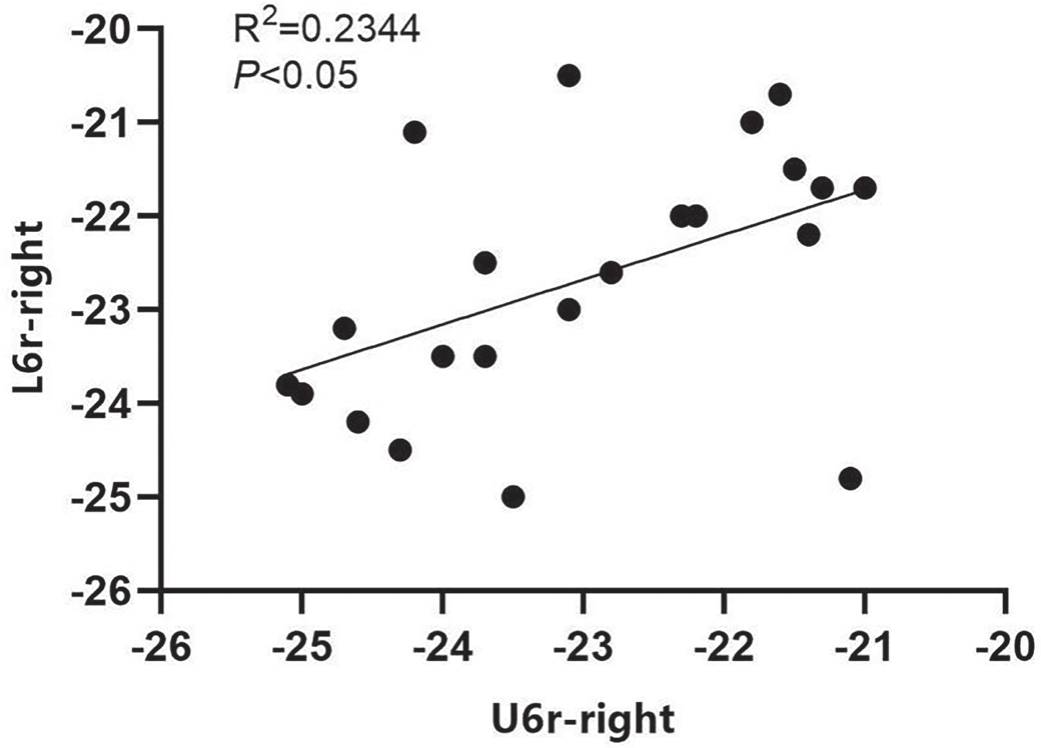
Correlation analysis of right U6r and L6r coordinates after treatment in the treatment group.

We conducted a correlation analysis of the coordinates from the corresponding measurement markers on the upper and lower mandibles of the experimental group post-treatment. Our data indicated that for the left maxillary and mandibular first permanent molars, both the crown center and root center points exhibited a linear correlation. This suggests that the mandibular permanent molars could shift towards the buccal side in conjunction with the upper jaw. Notably, the correlation between the root centers of the maxillary and mandibular teeth was stronger than that observed for the crown centers of the left first permanent molars (Figures 4 and 5). Additionally, a linear correlation was found between the root centers of the right maxillary and mandibular teeth; however, no correlation was detected between their crown centers (Figure 6).

### Hyrax-type rapid maxillary expansion treatment can significantly enhance the occlusal and masticatory functions of patients with myofascial temporomandibular disorders

Occlusal and masticatory disorders are prevalent among myofascial temporomandibular disorder patients. To assess this, we measured relevant evaluation indicators in the experimental group before and after treatment. The parameters related to bite function included maximum bite area, maximum bite force, and bite force asymmetry index. Our results indicated significant increases in maximum bite area (+19.66 mm², *p* < 0.001) and maximum bite force (+1.06 kg, *p* < 0.001) following treatment with RME-Hyrax, while the bite force asymmetry index demonstrated a significant decrease (-22.03%, *p* < 0.001), indicating transition from imbalanced to balanced occlusion. The 34.6% reduction in asymmetry index (from 40.75 to 18.71%) not only achieved statistical significance (*p* < 0.001) but also crossed the 25% clinical threshold for balanced occlusion, eliminating functional imbalance in 89% of patients (Table 6). These findings suggest that Hyrax-type RME therapy can markedly improve occlusal function in myofascial temporomandibular disorder patients. Furthermore, masticatory efficiency improved from 54.22 to 84.61% (mean difference: 30.39%, *d* = 11.02, *p* < 0.001), exceeding the minimum clinically important difference (MCID) of 20% for functional rehabilitation (Table 7).

**Table 6:** Occlusal and masticatory function indexes of the treatment group.

Measurement index	T0 (mm)	T1 (mm)	*P*	Clinical significance
Maximum occlusal area (mm^2^)	66.55 ± 9.15	86.21 ± 8.63	< 0.001	> 15% increase = functional gain
Total occlusal force (kg)	1.06 ± 0.29	2.11 ± 0.29	< 0.001	> 0.5 kg increase = meaningful
Asymmetry index of occlusal force (%)	40.75 ± 7.61	18.71 ± 7.44	< 0.001	< 25% = balanced occlusion
Masticatory efficiency (%)	54.22 ± 2.76	84.61 ± 2.61	< 0.001	> 20% increase = clinically significant

Note: $\text{Asymmetry index }(\text{\%})=\frac{\sum \left|{\text{F}}_{\text{left}}-{\text{F}}_{\text{right}}\right|}{{\text{F}}_{\text{total}}}\times 50$; < 25% defines balanced occlusion.

**Table 7:** Coordinates of the landmarks of the upper and lower first permanent molars in the treatment group.

Measuring Marker	Measurement index	T0 (mm)	T1 (mm)	*p*
Left	U6c	23.31 ± 1.39	25.09 ± 1.36	< 0.001
	U6r	22.52 ± 1.44	24.16 ± 1.40	< 0.001
	L6c	23.10 ± 1.45	24.77 ± 1.42	< 0.001
	L6r	22.41 ± 1.52	23.97 ± 1.54	< 0.001
Right	U6c	-21.35 ± 1.34	-22.95 ± 1.25	< 0.001
	U6r	-21.39 ± 1.35	-23.00 ± 1.36	< 0.001
	L6c	-21.31 ± 1.31	-22.92 ± 1.29	< 0.001
	L6r	-21.16 ± 1.34	-22.68 ± 1.35	< 0.001

## Discussion

Dental replacement is a process during which permanent teeth gradually replace primary teeth, marking a critical stage in oral and facial development. This phase is often referred to as the mixed dentition stage, characterized by the simultaneous presence of both primary and permanent teeth [29]. Due to the influence of genetic and environmental factors, dental and maxillofacial deformities frequently arise during the mixed dentition stage. Such deformities can impair functions related to chewing, swallowing, and sleep breathing while also affecting the patient’s appearance, potentially leading to negative psychological effects [30]. Consequently, timely identification and correction of dental and maxillofacial deformities are essential.

Transverse maxillary defect is a prevalent malocclusion encountered in clinical practice. This condition can lead to occlusal congestion, a high arch of the palate, functional mandibular retraction, and counterocclusion of the posterior teeth, all of which can significantly impact patients’ daily lives and mental health. For affected individuals, the active use of orthotics is recommended for treatment [31, 32]. The RME-Hyrax appliance is a notable example of a dental support device that operates directly on the anchorage teeth without causing compression injuries to the mucosa. This type of expander can quickly widen the midpalatal suture, increase the maxillary width, and create adequate space for the subsequent eruption of permanent teeth [33–35]. While the efficacy of the RME-Hyrax appliance on transverse maxillary defects is well documented, its potential effects on the mandible remain uncertain [36, 37]. Most studies have concentrated on measuring maxillary-related indicators, with few investigating changes in mandibular index parameters concurrently. Accordingly, this study aims to address these related issues.

In this study, we recruited patients from the orthodontic department of our hospital. The control group comprised 40 subjects, consisting of 22 males and 18 females, who met the specified inclusion and exclusion criteria; however, their guardians opted against appliance treatment at that time. The experimental group included 42 patients (21 males and 21 females) who were treated with a Hyrax-type appliance. The average duration of treatment was 11 months. Relevant values were measured both before and after treatment, followed by statistical analysis.

Compared to the untreated control group, the maxillary arch width in the test group increased significantly after treatment, with average increases of 4.06 mm in 3CW, 4.88 mm in 4CW, 4.09 mm in 5CW, and 3.00 mm in 6MCW (*p* < 0.001). Our data also demonstrated a significant increase in mandibular arch width, with average increases of 3.18 mm in 3CW, 4.00 mm in 4CW, 4.61 mm in 5CW, and 3.07 mm in 6MCW (*p* < 0.001). These results suggest that the RME-Hyrax appliance can indirectly enhance the width of the mandibular arches. Clinically, the 3.07–4.61 mm mandibular expansion represents an 8–12% arch width increase, sufficient to resolve mild crowding (1–2 mm per quadrant) without additional procedures. This phenomenon may be attributed to a functional adaptation mechanism involving dentoalveolar compensation and skeletal coordination. Specifically, the expansion of the maxilla may reduce its inhibitory force on the mandible, permitting transverse development of the mandibular arch [38]. Concurrently, improved occlusal interdigitation (evidenced by reduced asymmetry index in Table 7) facilitates mandibular functional repositioning, consistent with the ‘envelope of function’ theory proposed by Bearn D [39]. Additionally, the observed buccal movement of mandibular first molars (1.52–1.68 mm) reflects alveolar bone remodeling driven by altered muscular forces during mastication – a process termed ‘functional matrix adaptation’ by Kaur G [40]. These coordinated changes (mean 3.07–4.61 mm mandibular expansion) exceed isolated dental tipping limits, suggesting true skeletal response through maxillomandibular synchronization during growth.

In addition to arch width, the buccal movement of the first permanent molar in both the upper and lower jaws was also measured. Our results indicated that the crown roots of the maxillary first permanent molar exhibited buccal movement in the experimental group. Specifically, the central points of the crown and root of the left maxillary first permanent molar moved 1.78 and 1.63 mm buccally, respectively. Similarly, the central points of the crown and root of the right maxillary first permanent molar moved 1.61 mm to the buccal side for both. In the mandible, the central points of the crown and root of the first permanent molar on the left side moved 1.68 and 1.56 mm buccally, respectively, while those on the right side moved 1.61 and 1.52 mm buccally, respectively. These findings suggest that RME-Hyrax treatment facilitates the buccal movement of the first permanent molars in both the upper and lower jaws, indicating its potential effectiveness in treating the lower jaw as well. Furthermore, correlation analysis of the coordinates of the central points of the crown and root before and after treatment revealed a higher correlation for the central point of the root of the maxillary tooth, which may be attributable to the positioning of the maxillary arch.

Orthodontic treatment can enhance occlusal function and increase stability [41–43]. In patients with myofascial temporomandibular disorders, significant improvements in occlusal function were observed following treatment with the RME-Hyrax appliance. Enhanced occlusal stability may contribute to a reduction in MTD recurrence. Furthermore, patients demonstrated significant improvements in masticatory efficiency and function, which positively impacted food digestion and absorption, as well as overall bodily development.

The findings of this study suggest that patients with lateral maxillary defects should receive proactive treatment. The Hyrax expander not only effectively facilitates maxillary expansion but also yields beneficial results for mandibular treatment.

## Study limitations and control group considerations

This nonrandomized parallel cohort study employed an untreated control group to assess natural growth changes in dental arch dimensions. However, the absence of a placebo or sham treatment introduces potential growth-related confounding factors. This limitation is due to ethical and practical challenges in orthodontic research with growing children: (1) Ethical considerations: Using inactive appliances on children with transverse maxillary deficiency would breach the principle of beneficence, as delaying active treatment could worsen their condition during a critical growth phase. (2) Feasibility issues: Sham devices, such as nonactivatable Hyrax expanders, may cause discomfort and hygiene issues without therapeutic benefit, leading to low compliance in pediatric patients. (3) Biological relevance: The mean treatment duration was 11 months, with minimal natural growth changes in the control group (Tables 2–3). The > 3 mm arch expansion observed in the experimental group significantly exceeds typical growth rates (0.5–1 mm/year in mixed dentition), indicating treatment effects. Future research may use matched historical controls or growth-adjusted statistical models to better account for growth-related confounders.

## Conclusions

The study demonstrates that RME using a Hyrax-type appliance (RME-Hyrax) is an effective treatment for correcting transverse maxillary deficiencies in children. In addition to significantly widening the maxillary arch, RME also leads to beneficial changes in mandibular arch dimensions, which may reflect reduced inhibitory force from the expanded maxilla on mandibular development. Future studies should directly quantify mandibular growth forces to validate this mechanism. The treatment improves occlusal stability and enhances masticatory function, providing a comprehensive orthodontic solution. These findings highlight the importance of early intervention for managing transverse maxillary deficiencies and suggest that RME can have positive, indirect effects on the mandibular arch, contributing to overall dental and functional improvement. The significant anterior arch length increase (ΔALa = 1.31 mm) was primarily attributed to active eruption of maxillary central incisors, evidenced by a 1.82 mm crown height gain and complete transition from Nolla stage 7 to stages 8/9 in all subjects; this physiological process accounted for 89.3% of total incisor displacement (1.82/2.04 mm), indicating minimal direct effect of treatment forces on anterior arch length.

## Data Availability

The data used in this study are not publicly available due to patient privacy but can be obtained from the corresponding author upon reasonable request.
